# Editorial: Physiological Impacts of Global Warming in Aquatic Organisms

**DOI:** 10.3389/fphys.2022.914912

**Published:** 2022-05-09

**Authors:** I. Fernández, M. T. Mozanzadeh, Y. Hao, E. Gisbert

**Affiliations:** ^1^ Centro Oceanográfico de Vigo, Instituto Español de Oceanografía (IEO), Vigo, Spain; ^2^ South Iran Aquaculture Research Centre, Iranian Fisheries Science Institute (IFSRI), Agricultural Research Education and Extension Organization (AREEO), Ahwaz, Iran; ^3^ Institute of Hydrobiology, Chinese Academy of Sciences (CAS), Wuhan, China; ^4^ Aquaculture Program, Institut de Recerca i Tecnología Agroalimentaries (IRTA), La Rápita, Spain

**Keywords:** climate change, physiology, crustaceans, pico-phytoplankton, fish, metabolism, adaptative traits

Climate change is reshaping our planet. Warming surface waters, acidification, and deoxygenation are the most critical effects of climate change in aquatic environments. Increasing mean water temperatures modify species distribution, alters their basal metabolic rates, the occurrence and intensity of marine diseases, and the timing of pivotal biological events, among others. Ocean acidification results in physiological stress and inhibits the growth and calcification of endo- and exo-skeletons, while ocean deoxygenation, and particularly hypoxic events, may alter the distribution, aerobic scope, and survival of organisms (Reid et al., 2019). As climate change is projected to continue over this century and beyond, it is expected that the above-mentioned stressors will be intensified, further altering the structure, and functioning of marine ecosystems (Benedetti et al., 2021). Understanding and predicting the effects of climate change is one of the most pressing challenges in marine science, since this knowledge has an impact on fisheries, aquaculture, conservation, and applied ecology. Under this scenario, this Research Topic was conceived for updating and increasing the knowledge of ocean water rise on the biology and physiology of aquatic species, resulting in a Research Topic of four works on crustaceans, fish, and phytoplankton.

The study of Fusi et al. was designed to test the hypothesis that the metabolic levels of crabs were not only temperature-driven but were adjusted depending on the available dissolved oxygen. Thus, authors evaluated the respiratory rate in the mud crab *Thalamita crenata* under different temperatures (29–40°C) and correlated crab’s metabolic rate with their movements between habitats differing in daily oxygen levels (i.e., mangrove creeks and fringes, and seagrass meadows). Interestingly, authors showed how *T. crenata* could modulate their thermal response in a stringent dependency with water oxygen levels and confirmed that aerobic metabolism is not only regulated by temperature, but largely varied according with oxygen levels. For instance, when water was undersaturated, crabs’ metabolism was lowered, becoming temperature independent. In contrast, when oxygen availability was above full saturation and temperature caused changes in oxygen consumption rates, the increase of aerobic demand became fulfilled by water oxygen content, which extended the thermal tolerance of this species up to thermal extremes. These results showed the ability of *T. crenata* to modulate oxygen consumption rates, revealing an evolutionary adaptation of aquatic animals inhabiting these coastal habitats to daily broad oxygen variations, and explained their capacity to exploit a mosaic of different habitats with significant differences in oxygen and temperature profiles.

Lam et al. evaluated whether behavioural thermal limits in the porcelain crab *Petrolisthes cinctipes* varied due to their reproductive condition and its influence on population demographics under different thermal conditions. The combination of demographic, molecular, behavioural, and physiological measurements allowed the former authors to conclude that the distribution and demographics of *P. cinctipes* along the shore intertidal gradient depended on temperature and females’ reproductive condition. In particular, gravid crab females were more sensitive to warmer conditions, which indicated that as warming occurred, the most fecund individuals moved to cooler areas first, leaving warm edge populations with fewer reproductive individuals. For those females not avoiding the intertidal zone with warmer temperatures, a delay in reproduction may occur until temperatures cooled down. Authors also indicated that gravid females were more averse to habitats with high temperatures, since they had to invest a substantial amount of energy on brood care in addition to reproductive output, which placed constraints on their available energy budget for activity and fewer energy reserves available to tolerate thermal stress. This study showed how ectothermic animals adapt to microhabitat changes and their potential behavioural and reproductive adaptation to warming conditions derived from global climate change.

van der Walt et al. tested the thermal tolerance in blacktail seabream *Diplodus capensis*, a tropical species from Southeast Atlantic and Western Indian Ocean waters. Different cardiac indices were measured, using a micro heart rate logger, in fish exposed to different temperatures (13–31°C). A choice made for understanding the thermal tolerance of this species and its response to the predicted future increase in water temperatures, as well as its potential vulnerability to marine heat waves. After the individual analysis of twelve specimens, authors concluded that the suitable thermal habitat for adult *D. capensis* along the tropical edge of their distribution may be lost if mean summer temperatures increase beyond 28.3°C. In addition, predicted water warming in summer along the northern edge of this species range may result in distribution range contraction. This kind of physiological studies using biosensor technology are of special relevance since they are a reliable tool for evaluating individual responses to biotic and abiotic conditions (Calduch-Giner et al., 2022).

The study of Listmann et al. aimed to evaluate growth rates and metabolism of eight novel strains of pico-phytoplankton belonging to the genus *Ostreococcus* and understand how phytoplankton may evolve to global warming. These strains were obtained from two regions of the Baltic Sea differing in salinity and temperature. For this purpose, authors evaluated growth responses of *Ostreococcus* sp. under different temperatures (15 *vs*. 20°C), while authors characterized how growth changed in varying environments by quantifying the efficiency of carbon use *via* photosynthesis and respiration measurements. The study also evaluated how *Ostreococcus* sp. was able to grow on several organic carbon compounds considering again effects of changes in the thermal environment, evolutionary history, and the life cycle of this pico-phytoplanktonic species. This integrative study showed that strains of *Ostreococcus* sp. had an adaptive response to environmental changes due to differences in ecological variability and evolutionary history; in brief, strains from warmer and more variable areas showed higher growth rates and variable responses to temperature in comparison to those strains from colder and less variable regions. This is of relevance for modelling and understanding the response of phytoplankton to global warming (Harvey et al., 2022). Furthermore, authors also showed that inorganic and organic carbon sources were taken up in different quantities depending on the stage of the growth cycle and water temperature, in order to sustain their growth, fitness and adapt to changing conditions.

In conclusion, integrative studies in ectothermic organisms from tropical, temperate, and cold aquatic environments as those presented here, represent pertinent approaches for entangling how aquatic organisms will respond to climate change. These studies also proved how holistic studies and the integration of trait-based approaches allows a better understanding of how the rising of ocean water temperatures will drive patterns of geographic distribution, local abundance, and functional diversity of aquatic organisms.
